# The Legal and Ethical Dimensions of Hospital Visitation Bans in the COVID-19 Era

**DOI:** 10.3390/healthcare13030288

**Published:** 2025-01-31

**Authors:** Nicholas Lassi, Su Jiang, Yu Du

**Affiliations:** 1 School of Law, Southwest University of Political Science and Law, Chongqing 401120, China; luckynickphd@gmail.com; 2 Law School, Peking University, Beijing 100871, China; sujiang@pku.edu.cn; 3 Law School, Fudan University, Shanghai 200437, China

**Keywords:** patient visitation rights, public health legislation, emergency decrees, human rights law, healthcare regulation

## Abstract

**Background/Objectives:** The COVID-19 pandemic compelled countries worldwide to implement stringent visitation restrictions across hospitals, nursing homes, and long-term care facilities to mitigate viral transmission. While initially justified by the uncertainty surrounding the virus, these restrictions often lasted well beyond the acute stage of the pandemic, leading to substantial psychological and physical harm, particularly for older adults. This study assesses the effects of these controls and offers strategies to balance public health priorities with patients’ rights and psychological well-being during public health crises. **Methods:** An integrative review and comparative analysis of legislative measures and the psychological effects of visitation restrictions was undertaken. International and national visitation regulations and case studies were reviewed, and ethical frameworks were considered. **Results:** Our findings indicate that prolonged isolation due to extended visitation restrictions led to higher rates of anxiety, depression, and delirium among patients, creating ethical dilemmas for healthcare providers. Legal responses to this challenge varied globally. International human rights organizations called for policies bridging public health priorities with patients’ rights to family and caregiver support. Some U.S. states enacted proactive legislation to strengthen family visitation rights, while other jurisdictions lack these visitation protections. **Conclusions:** Prolonged visitation restrictions during the pandemic show the need for legislation integrating public health protections with in-person family and caregiver support. The ethical imperatives of limiting the psychological harm caused by healthcare isolation and the legislative solutions to protect public health and the psychological well-being of patients during health crises are discussed.

## 1. Introduction

The COVID-19 pandemic prompted worldwide visitation controls across hospitals, nursing homes, and other long-term care facilities to limit the spread of SARs-CoV-2. These measures were implemented to maintain controlled healthcare environments and protect patients and staff, particularly in the early stages, when the virus’s transmission pathways were not well understood [1,2,3]. The primary focus of these regulations was to protect high-risk groups, including older adults, individuals with pre-existing medical conditions, and those undergoing intensive medical treatments [4,5].

The scope and intensity of these visitation restrictions were unparalleled. Surveys show that during the pandemic’s first wave, 93 percent of U.S. hospitals and emergency departments adopted hospital-wide visitation bans, with only 58 percent allowing exceptions for end-of-life care [6]. Among the 70 largest U.S. hospitals, 49 had a general no-visitor policy [7]. Similar policies were implemented globally: in Taiwan, 11.8 percent of hospice wards banned visitors entirely, and among those allowing visitation, 43.3 percent limited visits to one hour per day [8]. In Germany, 10 out of 81 terminal patients died alone due to visitation restrictions [9]. In Canada, family presence at the time of death in intensive care units dropped dramatically from 86.6 percent pre-pandemic to 44.4 percent during the pandemic [10]. These restrictions also significantly reduced family involvement in critical treatment decisions [11].

Despite advances in COVID-19 knowledge and the widespread availability of personal protective equipment (PPE), many visitation restrictions persisted far past the initial outbreak [12]. A survey conducted over a year into the pandemic showed that 83 percent of U.S. hospitals allowed only one visitor per patient, and 69 percent maintained prohibitions on visitors for ICU patients with COVID-19 [5]. The continued application of these measures, even as safety protocols evolved, has raised significant ethical, legal, and medical questions.

The medical evidence supporting extended visitation bans remains inconclusive. Studies suggest minimal differences in transmission rates between facilities enforcing strict no-visitation policies and those permitting limited family visits [2,3,13,14]. Visitor-related transmission has also been identified as one of several potential pathways for hospital-acquired COVID-19 cases [15]. At the same time, the psychological toll of extended isolation was substantial, with patients experiencing an increased risk for anxiety, depression, and health deterioration [16].

Healthcare providers had the difficult task of balancing viral transmission control with protecting patients’ mental and emotional well-being. Balancing these conflicting interests represents significant challenges, provided the irregularities in prohibitions across jurisdictions, incomplete direction on ethical principles, and other related concerns such as the right to freedom of association [17]. Moreover, limited access to various kinds of patient support and assistance can have significant liability implications for healthcare providers [18].

Limitations detected within the literature were the catalyst for the development of this research. While previous discourse on visitation restrictions often centered on the psychological impact of these restrictions, this research devotes significant sections to legal, ethical, and policy analysis. In evaluating the legal precedents and policy positions in the U.S. and among international organizations, alternative perspectives to strict visitation restrictions were forwarded. By assessing how various governing entities managed public health protections and the legal and ethical duty to preserve human dignity and psychological well-being, the wider implications of various healthcare positions are conveyed.

This narrative review evaluated the legal and ethical components of healthcare visitation controls applied during COVID-19 and how they influenced patients and their families’ health and well-being. Provisions from the U.S. and international organizations were analyzed, such as Texas’s Essential Caregiver law, U.S. federal law, and the International Covenant on Civil and Political Rights (ICCPR). This study shows how legal and ethical questions and mental health are interconnected under these circumstances and how these connections can advise in the development of compassionate visitation rules during subsequent public health emergencies.

## 2. Materials and Methods

Integrative, thematic, and comparative methods were employed to investigate the implementation and consequences of family visitation restrictions in healthcare settings during the COVID-19 pandemic. An integrative method brought together legal, ethical, medical, and mental health elements within the material evaluated. For instance, the effects of visitation restrictions on mental health were merged with an analysis grounded in utilitarian and deontological ethical frameworks. This method also guaranteed that this study used quantitative results, such as measures of adverse health events among patients, and qualitative perspectives, such as protecting dignity in long-term care facilities.

A thematic approach detected and developed recurrent ideas across the material examined. The central themes involved the need for adaptive visitation guidelines, psychological care and advocacy in medical and long-term care environments, and balancing public health controls with mental health protections among individuals receiving care. This approach provided organized accounts of complicated topics while preserving clarity.

A comparative method showed differences and likenesses in visitation rules, their application, and results across populations. For instance, visitation rules during public health emergencies were compared among U.S. states and various international organizations, and the influence of visitation controls on different groups of patients was evaluated.

To guarantee consistency in the results, we performed searches across PubMed and Google Scholar databases to obtain a broad set of academic studies and reports from governments and international organizations. These databases were selected for their large medical, mental health, and law-related collections of academic materials.

The search procedure involved using singular and combinations of specific search terms to optimize relevance. The principal search terms included “healthcare/hospital visitation regulations”, “patient visitation rights”, “COVID-19”, “Healthcare emergencies”, “hospital isolation”, “psychological health”, and “international human rights guidelines”. Boolean operators were not used. Articles published between 2020 and 2024 were the primary focus, which corresponds to the pandemic timeframe. This period guarantees that the results involve current issues in healthcare visitation controls.

Standards for inclusion and exclusion were used to improve this study’s thoroughness and applicability. The criteria for inclusion were studies published in English, involving healthcare visitation guidelines during public health emergencies (with a focus on mental health, medical, and legal elements), centered on the psychological influence of visitation controls on various groups (e.g., children, older adults, and individuals with cognitive impairments), and those providing empirical evidence and policy perspectives. The criteria for exclusion were studies centered predominately on unconnected public health issues and those materials not subject to peer review, except when they were of substantial policy importance.

Various sub-sections within this study were developed from thematic and comparative methods that involved merging and evaluating resources corresponding to visitation controls during COVID-19. These segments were designed and organized to meet the review’s principal goal: to analyze the relationship between the law, healthcare visitation controls, and mental health issues while stressing their influence on end-of-life care and other vulnerable patients. For instance, the legislative actions and legal systems section examined how various legal mechanisms were devised to accommodate infection control and patients’ rights during COVID-19. The mental health and physical consequences section focused on the influence of visitation controls on patient and family well-being.

Two primary avenues of investigation were pursued: (1) a legal analysis of international and U.S. regulations governing healthcare visitation rights and (2) a review of empirical evidence on the psychological and physical effects of these visitation restrictions.

The legal analysis explored international guidelines and U.S. measures at the federal and state levels. Internationally, this study evaluated directives issued by organizations such as the United Nations (UN), the World Health Organization (WHO), and the European Centre for Disease Prevention and Control (ECDC). The core international human rights frameworks analyzed included the Universal Declaration of Human Rights (UDHR), the International Covenant on Civil and Political Rights (ICCPR), and the Convention on the Rights of Persons with Disabilities (CRPD). These materials represent the global standard in providing recommendations for balancing public health threats with the liberties of patients and their families.

Within the U.S., the legal analysis focused on federal policy, state laws, and regulatory measures that shaped visitation rules during the pandemic. Particular attention was given to state-level legislative responses in Florida, Texas, and North Carolina, which enacted strong family and caregiver visitation protections.

Empirical reports were gathered from various sources to understand the influence of visitation controls on patients. This review prioritized data on vulnerable populations, such as older adults and individuals with cognitive impairments. Central psychological outcomes examined included anxiety, depression, delirium, cognitive decline, and feelings of abandonment among patients. Physical health indicators, such as mobility and recovery rates, were also evaluated. The experiences of healthcare personnel were also taken into account, specifically their ethical considerations and psychological distress in administering strict visitation restrictions.

## 3. Results

### 3.1. International Healthcare Visitation and Human Rights Guidelines

Prominent international organizations, including the World Health Organization (WHO) and the United Nations (UN), set guidance on healthcare visitation policies, focusing on the principles of proportionality and non-discrimination. The WHO affirms the value of family connection for overall well-being, defining health as “a state of complete physical, mental, and social well-being” [19] (p. 1). During the COVID-19 pandemic, the WHO provided guidance on visitation protocols, wherein initial guidelines recommended stringent controls on hospital visitation, advising hospitals to “limit facility-based encounters” for safety reasons [20] (p. 8). However, subsequent updates advised policymakers to align restrictions with patients’ rights to family care.

Central human rights organizations generally promote healthcare visitation rights. During COVID-19, the UN Office of the High Commissioner for Human Rights (OHCHR) stressed that measures restricting family and caregiver access to patients must be necessary, proportionate, non-discriminative, and time-limited [21]. These measures were particularly concerned with vulnerable populations, such as individuals with disabilities, who require additional protections to prevent disruptions to support networks [21].

The Universal Declaration of Human Rights (UDHR) prohibits arbitrary interference with family life under Article 12, stating, “No one shall be subjected to arbitrary interference with his privacy, family, home, or correspondence”. Article 25 affirms the right to healthcare and well-being, reinforcing the necessity for reasonable visitation during public health crises [22]. Similarly, the International Covenant on Civil and Political Rights (ICCPR) protects family privacy under Article 17, offering legal remedies against excessive visitation restrictions [23] (Art. 2, Sec. 3 (a, b)).

The International Covenant on Economic, Social and Cultural Rights (ICESCR) advances the right to health, asserting that healthcare policies must not disproportionately harm patient welfare [24] (Art. 12). Additionally, the Convention on the Rights of Persons with Disabilities (CRPD) conveys the value of family involvement in supporting individuals with disabilities and prohibits arbitrary interference with family life [25] (Art. 22).

At the regional level, the European Convention on Human Rights (ECHR) protects family life under Article 8 [26]. The European Centre for Disease Prevention and Control (ECDC) has also issued recommendations balancing safety measures with patients’ psychological needs [27].

### 3.2. Federal Guidelines and State Laws in the United States

In the U.S., federal and state authorities issued patient safety and visitation provisions that evolved throughout the pandemic. At the federal level, the Centers for Medicare & Medicaid Services (CMS), in consultation with the Centers for Disease Control and Prevention (CDC), initially imposed strict visitation bans. In May 2020, the CMS directed nursing homes to prohibit nearly all visitors, with exceptions for compassionate care situations, such as end-of-life scenarios [28]. By September 2020, CMS eased these restrictions, allowing broader visitation under less stringent control measures [29]. These federal regulations, tied to Medicare and Medicaid funding, significantly influence healthcare policy (42 CFR § 482.13). Hospitals generally retain more independence in determining visitation policies than nursing homes, which are more tightly bound by CMS guidelines.

State governments exercised autonomy, leading to significant variability in visitation policies. States like New York and California implemented strict bans early in the pandemic that extended later than many states. For example, New York State’s early pandemic response included a total suspension of healthcare-related visitation except in end-of-life situations [30]. In March 2021, it permitted hospitals and nursing homes to allow limited family and caregiver visits [31,32].

Other states, such as Florida, Texas, North Carolina, and Arkansas, adopted more flexible, pro-visitation policies [33]. Emblematic of its general flexibility, Florida enacted its No Patient Left Alone Act in 2022, which guaranteed in-person visitation for essential caregivers, mandating at least two hours of daily visitation during public health emergencies [34]. North Carolina enacted similar legislation in 2021, requiring healthcare facilities to allow visitation to the fullest extent permitted under federal guidelines, with fines imposed for noncompliance [35] (Session Law 2021-171). Texas adopted a targeted approach with its “Essential Caregiver” law, focusing on long-term care facilities like nursing homes. Under this law, each patient may designate one essential caregiver whose visitation rights cannot be revoked except under specific, limited circumstances, such as major public health threats. Even then, suspensions are capped at 14 consecutive days and 45 days per year [36].

State legislation supporting visitation rights, such as Florida’s No Patient Left Alone Act, may face legal challenges related to federal guidelines and hospital policy conflicts. For instance, healthcare providers could argue that expanded visitation rights compromise safety, potentially leading to liability risks. However, constitutional protections of family autonomy, such as those affirmed in *Troxel v. Granville*, strengthen the argument for visitation as a fundamental liberty interest [37].

### 3.3. The Psychological and Physical Impact of Isolation in Healthcare Settings

Family members are integral to patient care, providing emotional support, advocacy, assistance with mobility, communication with staff, and facilitating decision-making during important transitions in care [3]. However, during the pandemic, their roles were significantly diminished, with families often relegated to being “insignificant others” or “insignificant caregivers” [38]. Patient isolation created a “shadow pandemic” of mental health challenges, increasing pre-existing vulnerabilities and fostering new ones [39,40].

Restricted family and caregiver access in healthcare settings prior to and during the COVID-19 pandemic has been associated with substantial psychological and physical disruptions for patients. The adverse effects of patient isolation include an increased risk of loneliness, anxiety, depression, and cognitive decline [1,41]. A Dutch survey of family members of nursing home residents found that 66 percent were concerned that their family member was experiencing heightened sadness during visitation restrictions. At the same time, 76 percent expressed concerns about their family members having increased feelings of loneliness during visitation restrictions [42]. A study conducted in China involving in-patients and their family members showed that during visitor restrictions, 42.44 percent of in-patients experienced anxiety, leading the researchers to conclude that “significant psychological impacts, such as feelings of loneliness and anxiety among hospitalized patients and their families, were evident” [43] (p. 8). An integrative review of research spanning from 1991 to 2022 reported that 40 percent of studies identified a positive relationship between the prevalence of family visitation and reduced incidences of depression [44].

In Japan, the incidence of delirium among emergency patients rose significantly from 1.8 percent pre-restrictions to 6.2 percent during restricted visitation [45]. Similarly, patients with acute cerebrovascular disease displayed a heightened risk of delirium during the pandemic restrictions, with the incidence of delirium at “6.3 percent during pandemic-associated absolute visitation restriction, 5.8 percent with limited visitation policy, and 5.1 percent with pre-pandemic visitation policy” [46] (p. 273). A large-scale study of more than 2000 critically ill patients with COVID-19 found that family visitation was linked to a 30 percent decrease in the likelihood of delirium among these patients [47]. Thilges et al. determined that during the COVID-19 visitor restrictions, the incidence of delirium among patients in a large medical system in the Midwest U.S. was 11.26 percent, a significant increase compared to the pre-pandemic rate of 9.28 percent [48]. A study by Qin et al. reported a 24 percent reduction in the risk of ICU delirium when family visitation was permitted [49], corroborating findings from a pre-pandemic meta-analysis linking restrictive visitation policies in ICUs to elevated anxiety and delirium rates [50].

Increased loneliness was reported among patients experiencing visitation restrictions [51]. Loneliness has been strongly correlated with depression [52], anxiety [53], suicidal ideation, and cognitive decline [54]. Physical health issues linked to loneliness include weakened immune responses [55], systemic inflammation [56,57], and cardiovascular problems [54,58,59].

Specific populations were disproportionately affected by visitation restrictions. Older adults in long-term care facilities experienced significantly higher rates of decline in physical functioning, including the reduced ability to perform activities of daily living (ADL) and deteriorating physical capabilities [60,61]. They also experienced a higher probability of mental health conditions such as negative mood [62], loneliness [63], stress, and memory loss [61].

Family and caregiver presence has been linked to improved physical recovery, including shorter hospital stays [64], fewer complications [65], reduced physical pain [1], lower fall rates [66], more timely medication administration, and better daily activity performance [67]. Pediatric patients particularly benefit from family involvement, which has been linked to better detection of medical errors and other adverse events [68,69]. This is because “Parents know their children best and often recognize when something is not right with their child and alert the nurse. This lack of parental presence during critical times, due to visitor restrictions, provides opportunity for negative outcomes” [70] (para. 6). Ultimately, the emotional and advocacy roles of family members have been shown to mitigate harm and facilitate recovery [67,71].

Restricted visitation policies imposed substantial psychological burdens on families and healthcare providers [72]. Restricted families of ICU patients reported significantly higher levels of anxiety, depression [73], and post-traumatic stress symptoms [74]. Families of pediatric patients in ICUs reported higher levels of distress under restricted visitation conditions [75,76,77]. For healthcare providers, particularly those in ICU and end-of-life care settings, the absence of family support has increased the propensity for emotional exhaustion, “compassion fatigue”, and stress, impeding the ability to deliver compassionate, high-quality care [78,79,80,81].

Not all studies indicated adverse outcomes from restricted visitation. Some research reported minimal or no significant associations between visitation policies and specific outcomes, such as ICU delirium [82,83], mechanical ventilation use, or mortality rates [49,64]. These results indicate that the negative influence of visitation controls may fluctuate in response to patient characteristics, healthcare conditions, and the standard of alternative care.

## 4. Discussion

### 4.1. Utilitarian and Deontological Ethics

Utilitarian and deontological ethical frameworks provide contrasting views for assessing healthcare visitation restrictions implemented during public health crises. Utilitarianism justifies these restrictions as necessary to prevent widespread infection, protect public health, and reduce overall mortality [84]. This perspective considers the emotional distress experienced by patients and families as a tolerable trade-off for the broader goal of maximizing social well-being. Such reasoning is derived from the principle of achieving “the greatest good for the greatest number” [85].

In contrast, deontology challenges the morality of these restrictions, asserting that individual rights and dignity must not be subordinated to collective goals. According to this ethical framework, denying patients physical access to their families—particularly during end-of-life care and for vulnerable populations—inflicts emotional anguish on those most at risk and represents an inherent violation of their dignity and moral worth [70]. Ethical duties, such as preserving family connections, should supersede broader utilitarian considerations, even in the context of significant public health risks [86,87]. This perspective conveys that the ends cannot justify the means if the means encroach on essential human rights.

These ethical principles illustrate a fundamental tension in healthcare visitation restrictions [88]. While utilitarianism prioritizes collective welfare [85], it risks marginalizing vulnerable individuals whose needs conflict with broader priorities. Conversely, deontology emphasizes safeguarding individual rights [84,86], even when doing so may complicate public health management. Visitation policy during public health crises must find a balance between these ethical principles.

### 4.2. The Need for a Unified Visitation Policy

The COVID-19 pandemic revealed the inadequacy of fragmented visitation policies, particularly in the U.S., where rules varied widely across states and healthcare facilities. This inconsistency in visitation policy created inequalities in access [89]. Applying uniform national and international rules could attend to these disparities, offer a lucid legal framework, and minimize disputes and litigation. These unified rules should recognize visitation as a fundamental part of patient care, particularly in nurturing psychological health, supporting recovery, and maintaining dignity. Expanding these guidelines into national and international standards could allay misunderstandings and inequalities.

International human rights guidelines, such as the Universal Declaration of Human Rights (UDHR) and the Convention on the Rights of Persons with Disabilities (CRPD), offer important direction, as they stress proportionality, non-discrimination, and the protection of family life. Unified rules could also adopt a flexible tiered approach, using data on viral transmission, hospital capacity, and patient vulnerability to adjust restrictions in real-time. Ultimately, standardization can safeguard equitable access to visitation and improve readiness for future crises by instituting clear and humane rules.

### 4.3. Supporting Psychological Health Through Family and Caregiver Presence

Studies consistently show the psychological and physical benefits of family and caregiver presence in healthcare settings. Regular visitation has been found to reduce the risk of anxiety, depression, and psychological distress while improving recovery outcomes and minimizing complications [1,65,90,91]. Beyond psychological support, family members and caregivers advocate for patients, facilitating communication between patients and healthcare providers [92,93]. This dual position—emotional support and practical advocacy—alleviates helplessness and stress for patients and their families [1,94].

For older adult patients, regular family visits have been linked to slower cognitive decline [1]. Interaction with familiar individuals can stimulate cognitive function and provide a sense of normalcy, which helps prevent delirium [45,47]. This is especially important in long-term care settings, where extended isolation heightens the risk of cognitive deterioration [95,96]. Family presence is also vital in end-of-life care, providing patients with emotional support and helping families in grieving [97,98,99].

Evidence suggests that, with appropriate infection prevention measures, the risks associated with patient visitation during COVID-19 were minimal [2,13,14]. For instance, the Ontario COVID-19 Science Advisory Table reports that family visits did not significantly contribute to COVID-19 transmission in hospitals with stringent prevention protocols [3]. These findings challenge the necessity of blanket visitation bans and suggest that policies should be recalibrated to include mental health and emotional considerations. More permissive visitation policies could enhance patient well-being during low-risk periods, while stricter controls may be warranted under high-risk conditions. However, even during severe public health crises, exceptions for end-of-life patients and vulnerable populations are necessary to uphold ethical responsibilities to dignity and human connection.

### 4.4. Optimizing Hospital Visitation Protocols for Public Health Crises

Public health crises like the COVID-19 pandemic have shown the necessity for safe visitation. Hospitals must invest in resources and infrastructure to accommodate visitation without jeopardizing safety. Designated visitation areas, increased staffing, and access to rapid testing kits can reduce risks while preserving the quality of care [100]. Social workers and healthcare coordinators can further support families by guiding them through visitation protocols and addressing their concerns [2].

While virtual communication methods have been utilized during public health crises, they are generally regarded as inadequate replacements for in-person support [101,102,103]. Virtual communication does offer a practical supplement when in-person visits are unfeasible, and it has shown some benefits. A survey conducted at a Toronto hospital during COVID-19 found that a virtual family visiting (VFV) program was positively received by patients and families [104]. Similar research has found that virtual visits increase feelings of family unity [105], reduce patient distress, and help orient patients experiencing delirium [106]. Physician–family virtual communication has also been shown to mitigate emotional strain among family members [107]. However, these virtual methods sometimes cannot cover the full psychological demands of patients and families and should generally remain secondary. For instance, there is evidence that video calls did not influence PTSD symptoms in ICU settings during COVID-19 [108], and ICU patients in France reported negative experiences with virtual communication [103].

Hospitals should prioritize flexible visitation policies for vulnerable populations, including patients with cognitive impairments, as these groups greatly benefit from in-person caregiver presence [109,110]. Policymakers and hospitals should also attend to inequities by establishing virtual communication options for families without access to virtual communication technologies, guaranteeing that resource disparities do not worsen the effects of patient isolation [111]. Investments in staff training are also necessary to maximize the effectiveness of in-person and virtual visitation in future crises. Studies have shown that time constraints, inadequate preparation, and low technological literacy among healthcare staff hinder the success of virtual visitation [3,112].

Patients and their families can take the initiative to moderate the negative influence of healthcare isolation. To preserve contact, they can advocate for video and other virtual communication applications in healthcare facilities. Also, they can appeal to government officials for legislative changes prioritizing compassionate visitation. The No Patient Left Alone Act, the Essential Caregiver law, and guidelines provided by international organizations like the United Nations are valuable sources when advancing change. Lastly, patients and their families can seek out counseling and other mental health options, either through in-person or video-based avenues, to attend to the influence of isolation and detachment.

### 4.5. Addressing Staff Concerns About Visitation Policies

Healthcare professionals, particularly nurses, have expressed concerns about more lenient family visitation, citing disruptions to workflow and increased workloads [113,114]. These concerns are valid, especially in high-pressure environments where resources are limited. However, it is equally important to recognize the extensive research showing the benefits of family involvement for patients, their families, and even healthcare teams [115].

## 5. Conclusions

Healthcare visitation restrictions imposed during the COVID-19 pandemic raised significant ethical and legal dilemmas related to patient mental health, showing the tension between controlling the spread of infectious diseases and safeguarding patients’ psychological well-being. While infection control measures remain paramount in public health crises, the detrimental effects of prolonged isolation on patients—such as higher anxiety, depression, cognitive decline, and moderated recovery outcomes—should not be overlooked. These issues present an opportunity to re-evaluate and refine visitation policies so that future strategies are evidence-based, compassionate, and proportionate to the risks involved.

Empirical data show that strict visitation controls during COVID-19 were linked with negative consequences involving patient mental health and human dignity, principally in long-term and end-of-life care. These controls often diverge from recognized ethical standards and global human rights guidelines, which stress the value of family and caregiver visitation in preserving psychological well-being. U.S. state-level provisions enacted during COVID-19 to protect in-person family and caregiver presence at healthcare facilities, developed in response to these challenges, can act as a framework for managing similar crises in the future.

Governments should enact laws guaranteeing that family presence at healthcare facilities is available for patients, even during times of crisis. During times of exceptional danger, policymakers should stress adaptability, permitting exceptions for vulnerable groups and end-of-life care with the integration of strict infection safeguards. Drawing on the views of Dugdale et al. [12], balanced visitation policies should be developed that address public health needs and the psychological well-being of patients. Adaptive strategies—such as emphasizing in-person visitation for vulnerable groups, employing virtual communication methods as a secondary option, and equipping healthcare sites with materials for safe visits—can guarantee that patients are never detached from family support.

Healthcare systems should incorporate ethical considerations into their policy platforms that strengthen in-person family and caretaker visitation protections. Healthcare staff should be trained in handling in-person visitation procedures to decrease disruptions in contact and advance family involvement. Regularly reassessing visitation strategies is also important to determine their effects on the patient’s mental and physical health. Data-driven evaluations can guide refinements to optimize strategies for the best outcomes for patients and healthcare facilities.

Future health emergencies may present unforeseen challenges, but safeguarding visitation rights should remain constant. Preserving access to at least one visitor, even during significant public health crises, will help maintain the human connections that reduce psychological distress and enhance recovery outcomes. Compassionate visitation rights protect the psychological health of patients and secure their dignity and humanity during difficult periods. By combining public health interests with compassion-based ethical principles, healthcare sites can be safe, supportive, and inclusive settings during future public health crises.

## Data Availability

No applicable.

## References

[1] Hugelius K., Harada N., Marutani M. (2021). Consequences of Visiting Restrictions during the COVID-19 Pandemic: An Integrative Review. Int. J. Nurs. Stud..

[2] Marmo S., Hirsch J. (2023). Visitors Not Welcome: Hospital Visitation Restrictions and Institutional Betrayal. J. Policy Pract. Res..

[3] Munshi L., Odutayo A., Evans G.A., Keresteci M., Drury J., Kain D., Johnstone J., Stall N.M., Barrett K., Slutsky A.S. (2021). Impact of Hospital Visitor Restrictions During the COVID-19 Pandemic.

[4] Fiest K.M., Krewulak K.D., Hiploylee C., Bagshaw S.M., Burns K.E.A., Cook D.J., Fowler R.A., Kredentser M.S., Niven D.J., Olafson K. (2021). An Environmental Scan of Visitation Policies in Canadian Intensive Care Units during the First Wave of the COVID-19 Pandemic. Can. J. Anesth./J. Can. Anesthésie.

[5] Marmo S., Milner K.A. (2023). From Open to Closed: COVID-19 Restrictions on Previously Unrestricted Visitation Policies in Adult Intensive Care Units. Am. J. Crit. Care.

[6] Lo A.X., Wedel L.K., Liu S.W., Wongtangman T., Thatphet P., Santangelo I., Chary A.N., Biddinger P.D., Grudzen C.R., Kennedy M. (2022). COVID-19 Hospital and Emergency Department Visitor Policies in the United States: Impact on Persons with Cognitive or Physical Impairment or Receiving End-of-life Care. J. Am. Coll. Emerg. Physicians Open.

[7] Jaswaney R., Davis A., Cadigan R.J., Waltz M., Brassfield E.R., Forcier B., Joyner B.L. (2022). Hospital Policies During COVID-19: An Analysis of Visitor Restrictions. J. Public Health Manag. Pract..

[8] Hsu Y.-C., Liu Y.-A., Lin M.-H., Lee H.-W., Chen T.-J., Chou L.-F., Hwang S.-J. (2020). Visiting Policies of Hospice Wards during the COVID-19 Pandemic: An Environmental Scan in Taiwan. Int. J. Environ. Res. Public Health.

[9] Schloesser K., Simon S.T., Pauli B., Voltz R., Jung N., Leisse C., van der Heide A., Korfage I.J., Pralong A., Bausewein C. (2021). “Saying Goodbye All Alone with No Close Support Was Difficult”—Dying during the COVID-19 Pandemic: An Online Survey among Bereaved Relatives about End-of-Life Care for Patients with or without SARS-CoV2 Infection. BMC Health Serv. Res..

[10] Cook D.J., Takaoka A., Hoad N., Swinton M., Clarke F.J., Rudkowski J.C., Heels-Ansdell D., Boyle A., Toledo F., Dennis B.B. (2021). Clinician Perspectives on Caring for Dying Patients During the Pandemic. Ann. Intern. Med..

[11] Renckens S.C., Pasman H.R., Jorna Z., Klop H.T., du Perron C., van Zuylen L., Steegers M.A.H., ten Tusscher B.L., van Mol M.M.C., Vloet L.C.M. (2024). Varying (Preferred) Levels of Involvement in Treatment Decision-Making in the Intensive Care Unit before and during the COVID-19 Pandemic: A Mixed-Methods Study among Relatives. BMC Med. Inform. Decis. Mak..

[12] Dugdale L.S., Esbensen K.L., Sulmasy L.S. (2023). Ethical Guidance on Family Caregiving, Support, and Visitation in Hospitals and Residential Health Care Facilities, Including During Public Health Emergencies: An American College of Physicians Position Paper. J. Gen. Intern. Med..

[13] Rhee C., Baker M., Vaidya V., Tucker R., Resnick A., Morris C.A., Klompas M. (2020). Incidence of Nosocomial COVID-19 in Patients Hospitalized at a Large US Academic Medical Center. JAMA Netw. Open.

[14] Wee L.E., Conceicao E.P., Sim J.X.-Y., Aung M.K., Venkatachalam I. (2021). The Impact of Visitor Restrictions on Health Care-Associated Respiratory Viral Infections during the COVID-19 Pandemic: Experience of a Tertiary Hospital in Singapore. Am. J. Infect. Control.

[15] Rickman H.M., Rampling T., Shaw K., Martinez-Garcia G., Hail L., Coen P., Shahmanesh M., Shin G.Y., Nastouli E., Houlihan C.F. (2021). Nosocomial Transmission of Coronavirus Disease 2019: A Retrospective Study of 66 Hospital-Acquired Cases in a London Teaching Hospital. Clin. Infect. Dis..

[16] Engström Å., Söderberg S. (2007). Receiving Power through Confirmation: The Meaning of Close Relatives for People Who Have Been Critically Ill. J. Adv. Nurs..

[17] McTernan E. (2023). Against Visitor Bans: Freedom of Association, COVID-19 and the Hospital Ward. J. Med. Ethics.

[18] Nioi M., Napoli P.E., Finco G., Demontis R., Fossarello M., d’Aloja E. (2021). Fear of the COVID-19 and Medical Liability. Insights from a Series of 130 Consecutives Medico-Legal Claims Evaluated in a Single Institution during SARS-CoV-2-Related Pandemic. Signa Vitae.

[19] World Health Organization Constitution of the World Health Organization (1946). Basic Documents, Geneva: World Health Organization.

[20] World Health Organization (2020). Maintaining Essential Health Services: Operational Guidance for the COVID-19 Context.

[21] Office of the High Commissioner for Human Rights (2020). COVID-19 Guidance.

[22] United Nations Office of the High Commissioner (1948). Universal Declaration of Human Rights.

[23] United Nations Office of the High Commissioner (1966). International Covenant on Civil and Political Rights.

[24] United Nations Committee on Economic, Social and Cultural Rights (2000). The Right to the Highest Attainable Standard of Health.

[25] Office of the High Commissioner for Human Rights (2006). Convention on the Rights of Persons with Disabilities.

[26] Council of Europe (1950). European Convention on Human Rights.

[27] European Centre for Disease Prevention and Control (2020). Infection Prevention and Control and Preparedness for COVID-19 in Healthcare Settings.

[28] Centers for Medicare & Medicaid Services (2020). CMS Announces New Measures to Protect Nursing Home Residents from COVID-19.

[29] Centers for Medicare & Medicaid Services (2020). Nursing Home Visitation—COVID-19.

[30] New York State Department of Health (2020). Health Advisory: COVID-19 Guidance for Hospital Operators Regarding Visitation.

[31] New York State Department of Health (2021). Interim Health Advisory: COVID-19 Updated Guidance for Hospital Visitation.

[32] New York State Department of Health (2021). Health Advisory: Revised Skilled Nursing Facility Visitation.

[33] Florida Health (2020). Florida Division of Emergency Management Issues Emergency Order to Lift Visitation Restrictions in Nursing Homes, Assisted Living Facilities and Other Long-Term Care Facilities.

[34] (2022). Florida Statutes Section 408.823. Legislature of the State of Florida.

[35] (2021). General Assembly of North Carolina Session Law 2021-171: Senate Bill 191. https://www.ncleg.gov/Sessions/2021/Bills/Senate/PDF/S191v5.pdf.

[36] (2021). Texas Health and Safety Code Chapter 260B. Right to Essential Caregiver Visits for Certain Residents.

[37] (2020). Troxel v. Granville 530 U.S. 57, 65. https://tile.loc.gov/storage-services/service/ll/usrep/usrep530/usrep530057/usrep530057.pdf.

[38] Berntzen H., Lind R., Alfheim H., Tøien K. (2023). Coping in Times of Disruption and Deprivation—Experiences of Family Members during COVID-19 Patients’ Critical Illness: A Qualitative Study. Nurs. Open.

[39] Cornforth M. (2022). The Shadow Pandemic: Three Reforms for the Post-Pandemic Mental Healthcare System.

[40] (2020). Nature Cancer Editorial Tackling the Shadow Pandemic. Nat. Cancer.

[41] Nielsen D.S., Hansen R.F., Beck S.H., Wensien J., Masud T., Ryg J. (2021). Older Patients’ Perspectives and Experience of Hospitalisation during the COVID-19 Pandemic: A Qualitative Explorative Study. Int. J. Older People Nurs..

[42] Wammes J.D., Kolk M.D., van den Besselaar M.J.H., MacNeil-Vroomen P.J.L., Buurman-van Es R.B.M., van Rijn P.M. (2020). Evaluating Perspectives of Relatives of Nursing Home Residents on the Nursing Home Visiting Restrictions During the COVID-19 Crisis: A Dutch Cross-Sectional Survey Study. J. Am. Med. Dir. Assoc..

[43] Xu Q., Ma J., Zhang Y., Rong Y., Lu S., Ge Q. (2024). The Effects of Visitor Restrictions on Inpatients and Family Visitors during the COVID-19 Pandemic: Insights from a Cross-Sectional Survey in China. BMC Public Health.

[44] Tan J.D.L., Maneze D., Montayre J., Ramjan L.M., Wang D., Salamonson Y. (2023). Family Visits and Depression among Residential Aged Care Residents: An Integrative Review. Int. J. Nurs. Stud..

[45] Kandori K., Okada Y., Ishii W., Narumiya H., Maebayashi Y., Iizuka R. (2020). Association between Visitation Restriction during the COVID-19 Pandemic and Delirium Incidence among Emergency Admission Patients: A Single-Center Retrospective Observational Cohort Study in Japan. J. Intensive Care.

[46] Hahn M., Gröschel S., Gröschel K., Uphaus T. (2023). Association of Delirium Incidence with Visitation Restrictions Due to COVID-19 Pandemic in Patients with Acute Cerebrovascular Disease in a Stroke-Unit Setting: A Retrospective Cohort Study. Gerontology.

[47] Pun B.T., Badenes R., Heras La Calle G., Orun O.M., Chen W., Raman R., Simpson B.-G.K., Wilson-Linville S., Hinojal Olmedillo B., Vallejo de la Cueva A. (2021). Prevalence and Risk Factors for Delirium in Critically Ill Patients with COVID-19 (COVID-D): A Multicentre Cohort Study. Lancet Respir. Med..

[48] Thilges S., Egbert J., Jakuboski S., Qeadan F. (2023). Associations between Delirium and SARS-CoV-2 Pandemic Visitor Restrictions among Hospitalized Patients. Public Health.

[49] Qin M., Gao Y., Guo S., Lu X., Zhu H., Li Y. (2022). Family Intervention for Delirium for Patients in the Intensive Care Unit: A Systematic Meta-Analysis. J. Clin. Neurosci..

[50] Nassar Junior A.P., Besen B.A.M.P., Robinson C.C., Falavigna M., Teixeira C., Rosa R.G. (2018). Flexible Versus Restrictive Visiting Policies in ICUs: A Systematic Review and Meta-Analysis*. Crit. Care Med..

[51] Engel F.D., da Fonseca G.G.P., Cechinel-Peiter C., Backman C., da Costa D.G., de Mello A.L.S.F. (2023). Impact of the COVID-19 Pandemic on the Experiences of Hospitalized Patients: A Scoping Review. J. Patient Saf..

[52] Czaja S.J., Moxley J.H., Rogers W.A. (2021). Social Support, Isolation, Loneliness, and Health Among Older Adults in the PRISM Randomized Controlled Trial. Front. Psychol..

[53] Dziedzic B., Idzik A., Kobos E., Sienkiewicz Z., Kryczka T., Fidecki W., Wysokiński M. (2021). Loneliness and Mental Health among the Elderly in Poland during the COVID-19 Pandemic. BMC Public Health.

[54] National Academies of Sciences, Engineering, and Medicine, Division of Behavioral and Social Sciences and Education, Health and Medicine Division, Board on Behavioral, Cognitive, and Sensory Sciences, Board on Health Sciences Policy, Committee on the Health and Medical Dimensions of Social Isolation and Loneliness in Older Adults (2020). Social Isolation and Loneliness in Older Adults: Opportunities for the Health Care System.

[55] Pourriyahi H., Yazdanpanah N., Saghazadeh A., Rezaei N. (2021). Loneliness: An Immunometabolic Syndrome. Int. J. Environ. Res Public Health.

[56] Van Bogart K., Engeland C.G., Sliwinski M.J., Harrington K.D., Knight E.L., Zhaoyang R., Scott S.B., Graham-Engeland J.E. (2022). The Association Between Loneliness and Inflammation: Findings from an Older Adult Sample. Front. Behav. Neurosci..

[57] Matthews T., Rasmussen L.J.H., Ambler A., Danese A., Eugen-Olsen J., Fancourt D., Fisher H.L., Iversen K.K., Schultz M., Sugden K. (2024). Social Isolation, Loneliness, and Inflammation: A Multi-Cohort Investigation in Early and Mid-Adulthood. Brain Behav. Immun..

[58] Bu F., Zaninotto P., Fancourt D. (2020). Longitudinal Associations between Loneliness, Social Isolation and Cardiovascular Events. Heart.

[59] Christiansen J., Lund R., Qualter P., Andersen C.M., Pedersen S.S., Lasgaard M. (2021). Loneliness, Social Isolation, and Chronic Disease Outcomes. Ann. Behav. Med..

[60] O’Caoimh R., O’Donovan M.R., Monahan M.P., Dalton O’Connor C., Buckley C., Kilty C., Fitzgerald S., Hartigan I., Cornally N. (2020). Psychosocial Impact of COVID-19 Nursing Home Restrictions on Visitors of Residents with Cognitive Impairment: A Cross-Sectional Study as Part of the Engaging Remotely in Care (ERiC) Project. Front. Psychiatry.

[61] Paananen J., Rannikko J., Harju M., Pirhonen J. (2021). The Impact of Covid-19-Related Distancing on the Well-Being of Nursing Home Residents and Their Family Members: A Qualitative Study. Int. J. Nurs. Stud. Adv..

[62] Noten S., Stoop A., De Witte J., Landeweer E., Vinckers F., Hovenga N., van Boekel L.C., Luijkx K.G. (2022). “Precious Time Together Was Taken Away”: Impact of COVID-19 Restrictive Measures on Social Needs and Loneliness from the Perspective of Residents of Nursing Homes, Close Relatives, and Volunteers. Int. J. Environ. Res. Public Health.

[63] Stolz E., Mayerl H., Freidl W. (2021). The Impact of COVID-19 Restriction Measures on Loneliness among Older Adults in Austria. Eur. J. Public Health.

[64] Goldfarb M.J., Bibas L., Bartlett V., Jones H., Khan N. (2017). Outcomes of Patient- and Family-Centered Care Interventions in the ICU: A Systematic Review and Meta-Analysis. Crit. Care Med..

[65] Duong J., Wang G., Lean G., Slobod D., Goldfarb M. (2024). Family-Centered Interventions and Patient Outcomes in the Adult Intensive Care Unit: A Systematic Review of Randomized Controlled Trials. J. Crit. Care.

[66] Shennan S., Coyle N., Lockwood B., DiDiodato G. (2024). Visitor Restrictions During the COVID-19 Pandemic and Increased Falls with Harm at a Canadian Hospital: An Exploratory Study. J. Patient Saf..

[67] Zeh R.D., Santry H.P., Monsour C., Sumski A.A., Bridges J.F.P., Tsung A., Pawlik T.M., Cloyd J.M. (2020). Impact of Visitor Restriction Rules on the Postoperative Experience of COVID-19 Negative Patients Undergoing Surgery. Surgery.

[68] Khan A., Coffey M., Litterer K.P., Baird J.D., Furtak S.L., Garcia B.M., Ashland M.A., Calaman S., Kuzma N.C., O’Toole J.K. (2017). Families as Partners in Hospital Error and Adverse Event Surveillance. JAMA Pediatr..

[69] Khan A., Furtak S.L., Melvin P., Rogers J.E., Schuster M.A., Landrigan C.P. (2016). Parent-Reported Errors and Adverse Events in Hospitalized Children. JAMA Pediatr.

[70] Smith M., VanGraafeiland B., McIltrot K. (2021). Visitor Restrictions During COVID-19: Ethical Considerations. American Nurse.

[71] Creutzfeldt C.J., Schutz R.E.C., Zahuranec D.B., Lutz B.J., Curtis J.R., Engelberg R.A. (2021). Family Presence for Patients with Severe Acute Brain Injury and the Influence of the COVID-19 Pandemic. J. Palliat. Med..

[72] Moss S.J., Krewulak K.D., Stelfox H.T., Ahmed S.B., Anglin M.C., Bagshaw S.M., Burns K.E.A., Cook D.J., Doig C.J., Fox-Robichaud A. (2021). Restricted Visitation Policies in Acute Care Settings during the COVID-19 Pandemic: A Scoping Review. Crit. Care.

[73] Rose L., Cook A., Onwumere J., Terblanche E., Pattison N., Metaxa V., Meyer J. (2022). Psychological Distress and Morbidity of Family Members Experiencing Virtual Visiting in Intensive Care during COVID-19: An Observational Cohort Study. Intensive Care Med..

[74] de Souza J.M.B., Miozzo A.P., da Rosa Minho dos Santos R., Mocellin D., Rech G.S., Trott G., Estivalete G.P.M., Sganzerla D., de Souza D., Rosa R.G. (2024). Long-Term Effects of Flexible Visitation in the Intensive Care Unit on Family Members’ Mental Health: 12-Month Results from a Randomized Clinical Trial. Intensive Care Med..

[75] Camporesi A., Abecasis F., Torres E.M., Zoia E., Izzo F., Ferrario S., Melloni E.M.T. (2022). The Parental Psychological Distress Caused by Separation from Their Critically Ill Child during the COVID-19 Pandemic: A Tale of Two Cities. Front. Pediatr..

[76] Krewulak K.D., Jaworska N., Lee L., Louis J.S., Dmitrieva O., Leia M.P., Doig C., Niven D.J., Parhar K.K.S., Rochwerg B. (2024). Impact of Restricted Family Presence during the COVID-19 Pandemic on Critically Ill Patients, Families, and Critical Care Clinicians: A Qualitative Systematic Review. BMC Health Serv. Res..

[77] Lee L.A., Foster J.R., Nikitovic D., Garros D., Ryan M.J., Moghadam N., Slumkoski C., Walls M., Curran J.A., Seabrook J.A. (2023). “We Aren’t Meant to Go Through the Hardest Parts of Our Lives Alone”: Family Experience with Restricted PICU Presence During the COVID-19 Pandemic. Crit. Care Explor..

[78] Azoulay E., Cariou A., Bruneel F., Demoule A., Kouatchet A., Reuter D., Souppart V., Combes A., Klouche K., Argaud L. (2020). Symptoms of Anxiety, Depression, and Peritraumatic Dissociation in Critical Care Clinicians Managing Patients with COVID-19. A Cross-Sectional Study. Am. J. Respir. Crit. Care Med..

[79] Malliarou M., Nikolentzos A., Papadopoulos D., Bekiari T., Sarafis P. (2021). ICU Nurse’s Moral Distress as an Occupational Hazard Threatening Professional Quality of Life in the Time of Pandemic COVID 19. Mater. Socio Medica.

[80] Shreffler J., Huecker M., Petrey J. (2020). The Impact of COVID-19 on Healthcare Worker Wellness: A Scoping Review. West. J. Emerg. Med..

[81] Zee M.S., Onwuteaka-Philipsen B.D., Witkamp E., Heessels B., Goossensen A., Korfage I.J., Becqué Y.N., Nierop-van Baalen C., van der Heide A., Pasman H.R. (2024). Distress among Healthcare Providers Who Provided End-of-Life Care during the COVID-19 Pandemic: A Longitudinal Survey Study (the CO-LIVE Study). BMC Palliat. Care.

[82] Rosa R.G., Falavigna M., da Silva D.B., Sganzerla D., Santos M.M.S., Kochhann R., de Moura R.M., Eugênio C.S., da Silva Riveiro H., Barbosa M.G. (2019). Effect of Flexible Family Visitation on Delirium Among Patients in the Intensive Care Unit. JAMA.

[83] Shinohara F., Unoki T., Horikawa M. (2022). Relationship between No-Visitation Policy and the Development of Delirium in Patients Admitted to the Intensive Care Unit. PLoS ONE.

[84] Chan P.S., Berg R.A., Nadkarni V.M. (2020). Code Blue During the COVID-19 Pandemic. Circ. Cardiovasc. Qual. Outcomes.

[85] Bentham J. (2007). An Introduction to the Principles of Morals and Legislation.

[86] Kant I. (1993). Grounding for the Metaphysics of Morals.

[87] Mandal J., Ponnambath D., Parija S. (2016). Utilitarian and Deontological Ethics in Medicine. Trop Parasitol..

[88] Tseng P.-E., Wang Y.-H. (2021). Deontological or Utilitarian? An Eternal Ethical Dilemma in Outbreak. Int. J. Environ. Res. Public Health.

[89] Weiner H.S., Firn J.I., Hogikyan N.D., Jagsi R., Laventhal N., Marks A., Smith L., Spector-Bagdady K., Vercler C.J., Shuman A.G. (2021). Hospital Visitation Policies During the SARS-CoV-2 Pandemic. Am. J. Infect. Control.

[90] Joo Y., Jang Y., Kwon O.Y. (2024). Contents and Effectiveness of Patient- and Family-centred Care Interventions in Adult Intensive Care Units: A Systematic Review. Nurs. Crit. Care.

[91] Sizoo E.M., Monnier A.A., Bloemen M., Hertogh C.M.P.M., Smalbrugge M. (2020). Dilemmas with Restrictive Visiting Policies in Dutch Nursing Homes During the COVID-19 Pandemic: A Qualitative Analysis of an Open-Ended Questionnaire with Elderly Care Physicians. J. Am. Med. Dir. Assoc..

[92] Bergerød I.J., Gilje B., Braut G.S., Wiig S. (2018). Next-of-Kin Involvement in Improving Hospital Cancer Care Quality and Safety–a Qualitative Cross-Case Study as Basis for Theory Development. BMC Health Serv. Res..

[93] Stenman L., Högberg L., Engström Å. (2022). Critical Care Nurses’ Experiences Caring for Patients When Relatives Were Not Allowed in the ICUs Due to COVID-19 Pandemic. SAGE Open Nurs..

[94] Haslund-Thomsen H., Thorup C.B., Laugesen B., Jørgensen L., Pedersen B., Voldbjerg S.L., Nielsen M.G., Jacobsen S., Kusk K.H., Albrechtsen M.T. (2023). Feeling Worried and Powerless: A Qualitative Interview Study of Relatives’ Experiences of the Collaboration with Patients and Nurses during COVID-19 Visiting Restrictions in Denmark. Nord. J. Nurs. Res..

[95] Lara E., Caballero F.F., Rico-Uribe L.A., Olaya B., Haro J.M., Ayuso-Mateos J.L., Miret M. (2019). Are Loneliness and Social Isolation Associated with Cognitive Decline?. Int. J. Geriatr. Psychiatry.

[96] Ren Y., Savadlou A., Park S., Siska P., Epp J.R., Sargin D. (2023). The Impact of Loneliness and Social Isolation on the Development of Cognitive Decline and Alzheimer’s Disease. Front. Neuroendocr..

[97] Bloomer M.J., Yuen E., Williams R., Hutchinson A.M. (2024). First and Final Farewells, Disrupted Family Connections and Loss: A Collective Case Study Exploring the Impact of COVID-19 Visitor Restrictions in Critical Care. Intensive Crit. Care Nurs..

[98] Feder S., Smith D., Griffin H., Shreve S.T., Kinder D., Kutney-Lee A., Ersek M. (2021). “Why Couldn’t I Go in To See Him?” Bereaved Families’ Perceptions of End-of-Life Communication During COVID-19. J. Am. Geriatr. Soc..

[99] Mateus M.J., Simões L., Ali A.M., Laranjeira C. (2024). Family Experiences of Loss and Bereavement in Palliative Care Units during the COVID-19 Pandemic: An Interpretative Phenomenological Study. Healthcare.

[100] Leiter R.E., Gelfand S. (2021). Even during a Pandemic, Hospitals Must Make Family Visits and Communication the Standard of Care. Stat.

[101] Jeong H., Choi Y., Kim H. (2023). Nonface-to-Face Visitation to Restrict Patient Visits for Infection Control: Integrative Review. Interact J. Med. Res..

[102] Forsberg T., Isaksson M., Schelin C., Lyngå P., Schandl A. (2024). Family Members’ Experiences of COVID-19 Visiting Restrictions in the Intensive Care Unit—A Qualitative Study. J Clin Nurs.

[103] Cattelan J., Castellano S., Merdji H., Audusseau J., Claude B., Feuillassier L., Cunat S., Astrié M., Aquin C., Buis G. (2021). Psychological Effects of Remote-Only Communication among Reference Persons of ICU Patients during COVID-19 Pandemic. J. Intensive Care.

[104] Dainty K.N., Seaton M.B., Molloy S., Robinson S., Haberman S. (2023). “I Don’t Know How We Would Have Coped without It”. Understanding the Importance of a Virtual Hospital Visiting Program During the COVID-19 Pandemic. J. Patient Exp..

[105] Xyrichis A., Pattison N., Ramsay P., Saha S., Cook A., Metaxa V., Meyer J., Rose L. (2022). Virtual Visiting in Intensive Care during the COVID-19 Pandemic: A Qualitative Descriptive Study with ICU Clinicians and Non-ICU Family Team Liaison Members. BMJ Open.

[106] Rose L., Yu L., Casey J., Cook A., Metaxa V., Pattison N., Rafferty A.M., Ramsay P., Saha S., Xyrichis A. (2021). Communication and Virtual Visiting for Families of Patients in Intensive Care during the COVID-19 Pandemic: A UK National Survey. Ann. Am. Thorac. Soc..

[107] Nordin A., Engström Å., Strömbäck U., Juuso P., Andersson M. (2024). Close Relatives’ Perspective of Critical Illness Due to COVID-19: Keeping in Touch at a Distance. Nurs. Open.

[108] Zante B., Erne K., Jeitziner M.-M. (2022). Video Calls Did Not Reduce PTSD Symptoms in Relatives during Restricted ICU Visits in the COVID-19 Pandemic. Sci. Rep..

[109] Welch M.L., Hodgson J.L., Didericksen K.W., Lamson A.L., Forbes T.H. (2022). Family-Centered Primary Care for Older Adults with Cognitive Impairment. Contemp. Fam. Ther..

[110] Wolff J.L., Roter D.L. (2011). Family Presence in Routine Medical Visits: A Meta-Analytical Review. Soc. Sci. Med..

[111] Haimi M. (2023). The Tragic Paradoxical Effect of Telemedicine on Healthcare Disparities—A Time for Redemption: A Narrative Review. BMC Med. Inf. Decis. Mak..

[112] Li M., Shi T., Chen J., Ding J., Gao X., Zeng Q., Zhang J., Ma Q., Liu X., Yu H. (2024). The Facilitators and Barriers to Implementing Virtual Visits in Intensive Care Units: A Mixed-methods Systematic Review. J. Eval. Clin. Pract..

[113] Wendlandt B., Kime M., Carson S. (2022). The Impact of Family Visitor Restrictions on Healthcare Workers in the ICU during the COVID-19 Pandemic. Intensive Crit. Care Nurs..

[114] Monroe M., Wofford L. (2017). Open Visitation and Nurse Job Satisfaction: An Integrative Review. J. Clin. Nurs..

[115] Sudai M. (2021). Not Dying Alone: The Need to Democratize Hospital Visitation Policies During COVID-19. Med. Law Rev..

